# Porcine circovirus type 2 associated reproductive failure in a specific pathogen free (SPF) piglet producing herd in Norway: a case report

**DOI:** 10.1186/s40813-017-0072-3

**Published:** 2017-10-24

**Authors:** M. Oropeza-Moe, A. J. Oropeza Delgado, Tore Framstad

**Affiliations:** 1 Norwegian University of Life Sciences (NMBU), Faculty of Veterinary Medicine, Department of Production Animal Clinical Sciences, Campus Sandnes, Sandnes, Norway; 2 Fiskå Mølle AS, Tau, Norway; 3 Norwegian University of Life Sciences (NMBU), Faculty of Veterinary Medicine, Department of Production Animal Clinical Sciences, Campus Adamstuen, Oslo, Norway

**Keywords:** Gilt, PCV2, Delayed farrowing, Mammary development, Mammary involution, Reproductive failure

## Abstract

**Background:**

Since 1999, scientists have published evidence of transplacental infection by porcine circovirus type 2 (PCV2) and reproductive failure in pigs. Affected herds have frequently been start-up herds, either naïve or with a high proportion of PCV2 susceptible gilts. Here, delayed farrowing in non-vaccinated gilts was observed in a commercial specific pathogen free (SPF) herd. Mummified fetuses and stillborn piglets recovered from these gilts were PCV2 positive.

**Case presentation:**

The case herd was a self-recruiting, piglet producing unit of 240 sows. After detecting livestock associated methicillin resistant *Staphylococcus aureus* (LA-MRSA, CC398), stamping out was imposed by the authorities. An SPF herd was re-established and all dams were vaccinated against PCV2 until the farmer decided to exclude this vaccine. The first non-vaccinated batch consisted of 76% gilts. Here, one gilt showed signs of impending farrowing. This gilt was slaughtered three to four weeks after the expected farrowing date without having expelled any uterine contents. In the subsequent batch consisting of 79% gilts, three gilts showed similar clinical signs. Delayed farrowing was observed in two of these gilts and the uterine contents from the third gilt were recovered at the abattoir. Mummified fetuses and stillborn piglets were recovered from all three gilts. High levels of PCV2 DNA (>10^7^ viral genomic copies/ 500 ng tissue) were found in myocardial samples by real-time PCR analysis. One myocardial sample submitted for immunohistochemical (IHC) analysis showed moderate amounts of PCV2 antigen. In the subsequent batch consisting of 77% gilts, several weak-born piglets were seen across different litters.

**Conclusions:**

This case report describes an apparent link between in utero PCV2 infection, pre partum nest-building behaviour, mammary development and delayed farrowing. To date, no reports have described imminent signs of farrowing and delayed farrowing as clinical signs in conjunction with transplacental PCV2 infection in Norway. Reinitiation of PCV2 vaccination was strongly advised in this herd due to recent depopulation and repopulation and the high proportion of gilts. Vaccination was effective because no further cases have occurred since.

## Background

Circoviruses are host specific viruses [1]. *Porcine circovirus type 1* (PCV1) and *porcine circovirus type 2* (PCV2) are acknowledged as the non-pathogenic and pathogenic types in swine, respectively [2]. PCV2 has been associated with a number of conditions collectively known as porcine circovirus diseases (PCVD). PCV2 has been suggested to play a role in the so-called porcine respiratory disease complex (PRDC), enteritis, porcine dermatitis and nephropathy syndrome (PDNS) and proliferative and necrotizing pneumonia (PNP) [3, 4]. A Canadian report published in 1999 was the first to describe PCV2 involvement in reproductive failure in swine [5]. Since then, several reports have documented PCV2-associated reproductive failure [6–19]. Affected herds have frequently been start-up herds in which the number of susceptible gilts is high or PCV2 seronegative herds [3, 5, 20]. Typical clinical signs associated with PCV2-associated reproductive failure are mummified, stillborn and weak-born piglets along with the macroscopical pathological findings including cardiomyopathy, pulmonary edema, hepatomegaly, and ascites [5, 8]. The most consistent microscopic changes include myocardial degeneration, fibrosis, and nonsuppurative to necrotizing or fibrosing myocarditis [5, 8, 21]. These changes are due to an apparent PCV2 tropism for fetal myocardiocytes, which diminishes with gestational age [22]. In the later stages of gestation, increased levels of PCV2 can be detected in lymphoid organs [21]. Clinical history, necropsy findings, histopathological and viral examination of fetal tissue samples demonstrating characteristic lesions and presence of PCV2 are necessary for the confirmative diagnosis of PCV2-associated reproductive failure.

Numerous laboratories have established methods for PCV2 DNA or antigen detection, e.g. real-time PCR or immunohistochemical (IHC) analyses. Clinical presentation of abortion, mummified fetuses and stillbirths in swine must be distinguished from other diseases caused by *porcine reproductive and respiratory syndrome virus* (PRRSV), *porcine parvovirus* (PPV), *pseudorabies virus, porcine enterovirus* (PEV) *and encephalomyelitis virus* (EMCV) [2, 23].

Vaccines against PCV2 infection are quite effective [24, 25] and vaccines marketed for sows exist (Circovac®, Merial, Lyon, France; Ingelvac CircoFLEX®, Boehringer Ingelheim Vetmedica GmbH, Ingelheim/ Rhein, Germany). The elected timepoint of dam vaccination can influence the antibody levels in piglets [26].

Information regarding transplacental PCV2 infection in conjunction with imminent signs of parturition (nest-building activity and mammary development) or delayed farrowing in gilts is limited [5, 27].

The aim of this case report is to describe the apparent correlation between transplacental PCV2 infection, mammary development and nest-building activity prior to the expected farrowing date and subsequently mammary involution and delayed farrowing (> 118 days) in a Norwegian commercial piglet producing SPF herd.

## Case presentation

The case herd was a self-recruiting, commercial piglet producing herd of 240 sows.

It was diagnosed with livestock associated methicillin resistant *Staphylococcus aureus* (LA-MRSA, C398) in April 2014. Norway has a national strategy for LA-MRSA including a search-and-destroy policy [28]. Therefore, the farmer was imposed stamping out the entire herd of 2550 pigs. The farm was decontaminated during the autumn of 2014 and the farmer decided to reestablish a closed SPF breeding herd. Landrace x Yorkshire gilts (210 days of age) were bought from one SPF breeding unit (free of *Actinobacillus pleuropneumoniae, Pasturella multocida, Mycoplasma hyopneumoniae, Brachyspira hyodysenteriae and Brachyspira pilosicoli)* until late autumn 2015. The farmer conducted insemination with Landrace semen (Norsvin SA, Hamar, Norway) and applied transabdominal ultrasound for determination of pregnancy. The herd had 1 gestation unit, 3 farrowing units and 3 weaner units. In the gestation unit, gilts and sows were kept in separate pens. The gestation unit was not washed and disinfected regularly. The farrowing units were washed and disinfected between every two to three batches. Every 7 weeks, around 70 sows farrowed.

Sows and gilts were fed standard gestation (Avlsfôr, Fiskå Mølle, Norway) and lactation diets (Opti Lakta, Fiskå Mølle, Norway). The gilts and sows were vaccinated against *Escherichia coli (*Neocolipor®, Merial Norden A/S, Denmark), PCV2 (Circovac®, Merial Norden A/S, Denmark), *Porcine parvovirus and Erysipelothrix rhusiopathiae* (Parvoruvax®, Merial Norden A/S, Denmark) according to the manufacturers recommendations. The farmer ceased to vaccinate dams against PCV2 in October 2015 since no beneficial effects on the overall production were perceived.

**Fig. 1:**
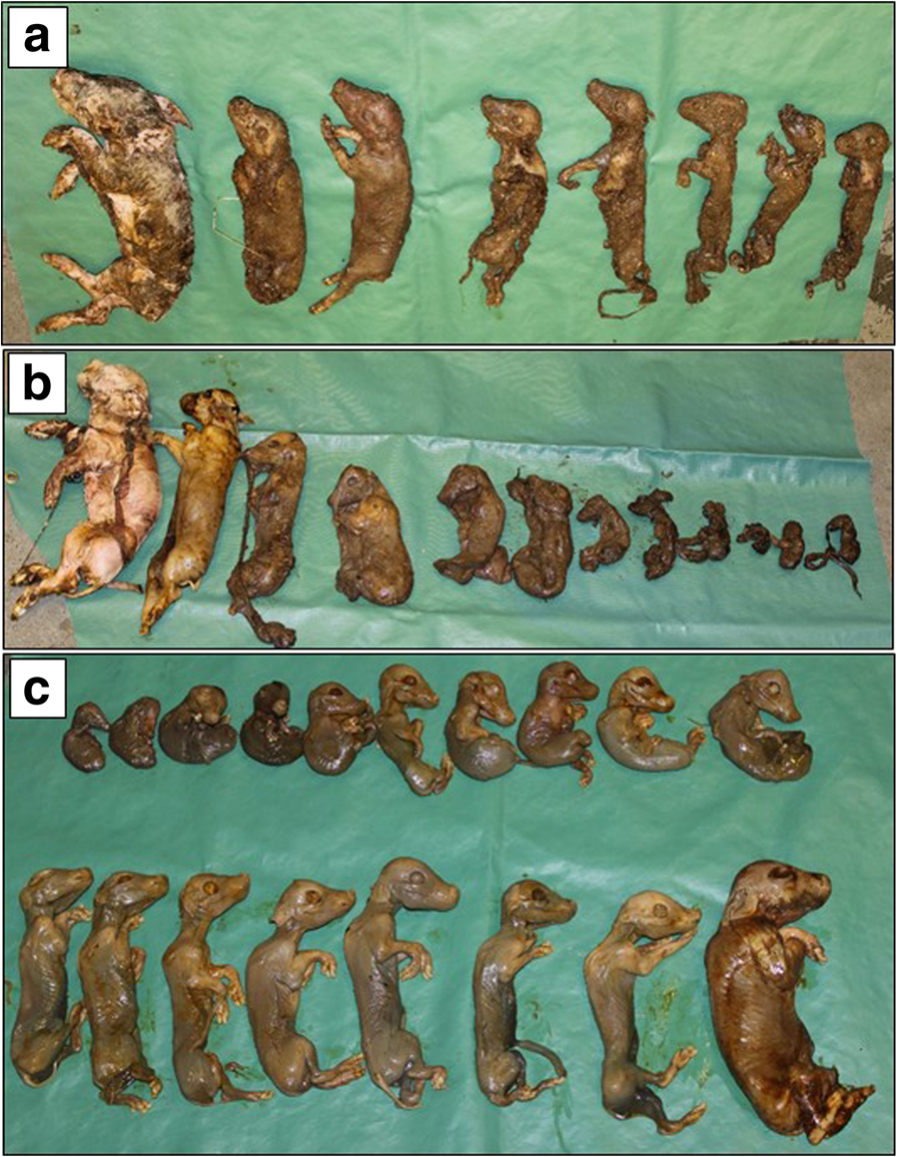
Mummified fetuses and stillborn piglets were submitted for post mortem examination. Two of the litters (**a** and **b**) were recovered by the farmer in the farrowing pens where gilts showing signs of imminent farrowing (nest building activity and mammary development) farrowed at a delayed stage (> 118 days of gestation). One of the litters (**c**) was recovered after the gilt was slaughtered at the abattoir. The uterus with contents was delivered to NMBU for sampling

In the first non-vaccinated batch consisting of 17 sows and 53 gilts (76% gilts), one gilt showed nest-building activity and mammary development without farrowing when expected. The gilt was slaughtered between three and four weeks after the expected farrowing date. The uterine contents were not examined. In the subsequent batch consisting of 15 sows and 55 gilts (79% gilts), three gilts showed similar clinical signs. Mummified fetuses and stillborn piglets (Litter A and B, Fig. 1a and b) were found in the pens approximately 20 and 24 days after the expected farrowing dates, respectively. Post mortem examination was conducted at the Norwegian University of Life Sciences (Sandnes, Norway). The weight/ crown-rump length (CRL) varied from 152 g/ 16 cm to 1380 g/ 29 cm in Litter A and from 31 g/ 9 cm to 1455 g/ 30 cm in Litter B. At the abattoir, 18 mummified fetuses were recovered from the remaining gilt (Litter C, Fig. 1c) sent to the abattoir 25 days after the expected farrowing date. Here, the weight/ CRL varied from 26 g/ 7 cm to 551 g/ 22 cm. In the subsequent batch consisting of 16 sows and 55 gilts (77% gilts), the number of weak born piglets across several gilt litters was high, but no exact numbers of weak born piglets across different litters were documented. However, 19 out of 20 piglets in one particular litter died within the first 24 h after birth. Unfortunately none of these weak born piglets were submitted for post mortem examinations. Udder involution was observed in all affected gilts around one week after the expected farrowing date.

**Fig. 2:**
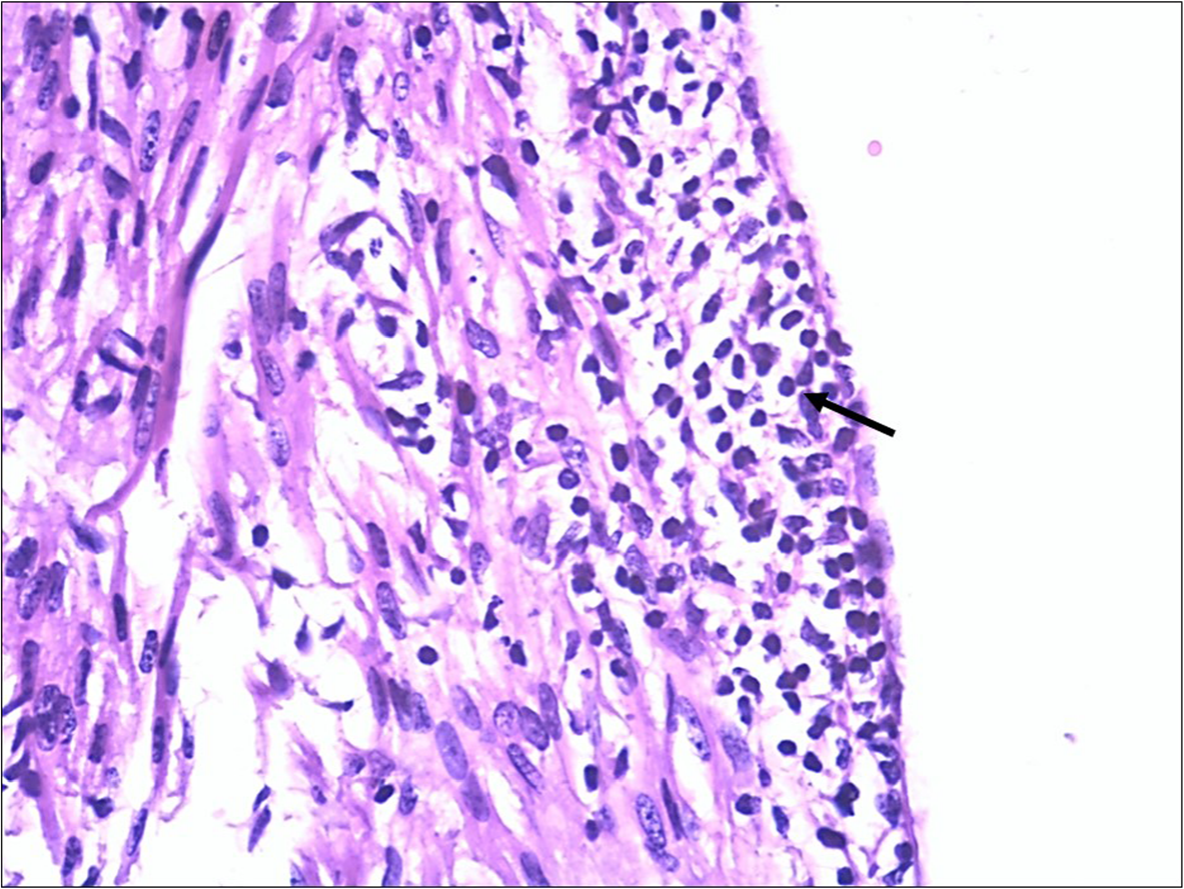
Myocardial inflammatory changes characterized by infiltrating mononuclear cells (arrows). Hematoxylin and eosin (HE) (magnification × 40)

Myocardial samples from two stillborn piglets representing Litter A and B were considered suitable for both histopathological and IHC analyses whereas the myocardium of the remaining mummified fetuses showed signs of advanced autolysis. The samples were processed by the DTU National Veterinary Institute (Copenhagen, Denmark). The myocardial samples from one piglet (Litter B) showed focal, subendocardial infiltrations of mononuclear cells (Fig. 2) and PCV2 capsid antigen was detected within myocytes with a non-commercial (F217) monoclonal antibody.

Bacteriological examinations of thoracic and abdominal organs (sheep blood agar, 37 °C, 5% CO_2_, 48 h) from all mummified and stillborn fetuses were negative.

High levels of PCV2 DNA (>10^7^ viral genomic copies/ 500 ng tissue) were detected by real-time PCR in nine myocardial samples from three piglets from litters A, B and C, respectively. Real-time PCR was conducted by the DTU National Veterinary Institute (Copenhagen, Denmark). No *Porcine teschovirus* (PTV) was detected in pulmonary samples from the three litters by real-time PCR [29]. This analysis was performed by the Instituto Nacional de Investigación y Technología Agraria y Alimentaria (INIA, Madrid, Spain). Real-time PCR analyses for *Porcine Parvovirus* (PPV) and *Porcine Reproductive and Respiratory Syndrome Virus* (PRRSV) on pooled liver samples (five from each litter) and pleural fluid from one piglet per litter, respectively, were negative (DTU National Veterinary Institute, Copenhagen, Denmark).

Serum samples from three randomly selected gilts and sows in the affected batch consisting of 79% gilts were analysed serologically. Antibody levels against *Leptospira icterohemorrhagiae, Chlamydia abortus*, EMCV or PEV were negative.

Based on the clinical signs, post mortem examinations and real-time PCR results, the farmer was advised to reinitiate vaccination against PCV2. All dams have been vaccinated with Circovac® since, and no similar cases have occurred.

## Discussion

There was an apparent correlation between PCV2 associated reproductive failure in non-vaccinated gilts and clinical signs of imminent parturition (mammary development and nest building activity), as well as mammary involution and delayed farrowing in the case herd. Two of the affected gilts expelled their PCV2 positive uterine contents three and four weeks after expected farrowing date. Additionally, two affected gilts were sent to the abattoir without having expelled the uterine contents. Mummified fetuses were recovered from one of these gilts and the fetuses were confirmed PCV2 positive. To the Authors knowledge, the correlation between transplacental PCV2 infection and the abovementioned clinical signs has been described twice previously [5, 27]. No reports from Norway have been published, describing this apparent link.

The criteria for diagnosis of PCV2 associated reproductive failure proposed by Segalés et al. were fulfilled in the case herd, including: (1) clinical signs which can include increased numbers of mummified fetuses, stillborns or weak-born pigs, (2) microscopic lesions within fetal tissues (heart or lymphoid tissues) and (3) PCV2 antigen or DNA within fetal tissues [4].

Dams carrying mummies and/ or stillborn piglets may farrow prematurely, at the expected time or later than expected time of farrowing [30]. If all fetuses die, the return to normal ovarian cyclicity may fail due to persistent corpora lutea [31]. If infection occurs during late gestation (70–115 days of gestation), the hormonal influence on pregnancy may remain and typical signs of impending parturition, like mammary development, may appear. All affected gilts in the case herd showed mammary development close to the expected farrowing time. Mammary involution occurred around one week after the expected farrowing time, a simple consequence of unsuckled mammary glands [32].

Microscopic myocardial lesions including infiltration of mononuclear cells were found along with PCV2 antigen identified by IHC. However, only one of two submitted samples could identify PCV2 by IHC. Fetal gross lesions are not always present following in utero PCV2 infection [33, 34]. Therefore, it may be challenging to select a representative sample for IHC staining. In the submitted samples, the cellular morphology of the labelled cells was often disrupted due to autolysis of the tissue. In this case, the detection of high levels of PCV2 DNA (>10^7^ viral genomic copies/ 500 ng tissue) by real-time PCR in fetal samples was essential for etiology determination.

The actual route of transmission remains unknown. Unfortunately none of the numerous weak-born piglets born in the third affected batch were submitted for post mortem examination. Based on previous reports, there is reason to believe that the clinical signs displayed by these piglets could very well be due to PCV2 infection [5, 6, 8, 35–37].

No further cases occurred after reinitiating vaccination against PCV2. Our assumption is that the unvaccinated gilts were PCV2 naïve and infected during pregnancy. The different sizes of mummificated fetuses indicate PCV2 infection of the affected gilts between 40 and 70 days of pregnancy [13, 38].

Serological testing for *Leptospira icterohaemorrhagica, Chlamydia abortus*, EMCV and *Porcine enterovirus group 1* antibodies was negative. Possible co-infections with other reproductive failure associated pathogens seem highly unlikely since all dams remained healthy until gilts started showing signs of reproductive failure.

The reintroduction of vaccination with Circovac® was effective since no further cases have occurred in the herd. Although PCV2 associated reproductive failure has been reported in PCV2 seropositive dams [39] and PCV2 vertical transmission has been shown to occur in PCV2-vaccinated dams [34], the correct timing of vaccination of sows and gilts can reduce viremia in the dam.

## Conclusions

This is the first case report to describe an apparent association between transplacental PCV2 infection in commercial SPF gilts and imminent signs of farrowing as well as delayed farrowing in Norway. PCV2 was confirmed in fetuses from three litters showing macroscopic and microscopic findings compatible with an in utero PCV2 infection. No further cases have occurred since reinitiating vaccination against PCV2, indicating a protective effect on PCV2 associated reproductive failure in the case herd.

## Abbreviations

DTU: National Veterinary Institute
EMCV: *encephalomyelitis virus*
IHC: Immunohistochemical; livestock associated methicillin resistant *Staphylococcus aureus*
INIA: Instituto Nacional de Investigación y Technología Agraria y Alimentaria
LA-MRSA: Livestock-associated methicillin resistant Staphylococcus aureus
NMBU: Norwegian University of Life Sciences
PCR: Polymerase chain reaction
PCV1: *Porcine circovirus type 1*
PCV2: *Porcine circovirus type 2*
PCVD: Porcine circovirus diseases
PEV: *Porcine enterovirus*
PPV: *Porcine parvovirus*
PRRSV: *Porcine reproductive and respiratory syndrome virus*
PTV: *Porcine teschovirus*
SPF: Specific pathogen-free
